# Ti_4_O_7_/g-C_3_N_4_ Visible Light Photocatalytic Performance on Hypophosphite Oxidation: Effect of Annealing Temperature

**DOI:** 10.3389/fchem.2018.00037

**Published:** 2018-03-01

**Authors:** Wei Guan, Gaoge Sun, Lei Yin, Zhenghua Zhang, Shichao Tian

**Affiliations:** ^1^Chongqing Key Laboratory of Environmental Materials and Remediation Technologies, Chongqing University of Arts and Sciences, Chongqing, China; ^2^Department of Chemistry, Tsinghua University, Beijing, China; ^3^Heibei Yinfa Meifute Environmental Engineering Co., Ltd.,, Shijiazhuang, China; ^4^Research Institute of Environmental Engineering and Nano-Technology, Graduate School at Shenzhen, Tsinghua University, Shenzhen, China; ^5^Shenzhen Environmental Science and New Energy Technology Engineering Laboratory, Tsinghua-Berkeley Shenzhen Institute, Shenzhen, China

**Keywords:** graphitic carbon nitride, sub-stoichiometric titanium oxides, hypophosphite, hydroxyl radicals, superoxide anion radicals

## Abstract

The oxidation of hypophosphite to phosphate is the key to recover the phosphorus resource from the hypophosphite wastewater. In the present work, Ti_4_O_7_/g-C_3_N_4_ composites were synthesized at two different temperatures (100 and 160°C) and their performance on photocatalytic oxidation of hypophosphite under visible light irradiation and the corresponding mechanism were evaluated. A hydrolysis method using g-C_3_N_4_ and Ti_4_O_7_ was applied to synthesize the Ti_4_O_7_/g-C_3_N_4_ composites with their hybrid structure and morphology confirmed by X-ray diffraction (XRD), scanning electron microscopy (SEM), and X-ray photoelectron spectra (XPS). The annealing temperature significantly affected the photocatalytic performance of Ti_4_O_7_/g-C_3_N_4_ that the 160-Ti_4_O_7_/g-C_3_N_4_ composite (fabricated at 160°C) showed the highest oxidation efficiency of hypophosphite of 81% and the highest photocatalytic oxidation rate of 0.467 h^−1^ comparing with the 100-Ti_4_O_7_/g-C_3_N_4_ composite (fabricated at 100°C) and pure g-C_3_N_4_. The enhanced photocatalytic performance of 160-Ti_4_O_7_/g-C_3_N_4_ could be ascribed to the effective charge separation and enhanced photoabsorption efficiency. Additionally, electron spin resonance (ESR) results showed that hydroxyl radicals and superoxide anion radicals were mainly responsible to the oxidation of hypophosphite with superoxide anion radicals accounting for a more significant contribution. Moreover, Ti_4_O_7_/g-C_3_N_4_ photocatalysts showed the remarkable stability in the repetitive experiments.

## Introduction

Hypophosphite is commonly used as a reducing agent in metallurgy industries especially in the processes of plating and surface finishing thereby generating large amounts of hypophosphite wastewater (Bulasara et al., 2011; Li et al., 2015). The hypophosphite contaminant should be further treated before being discharged into the river or lake, because it may lead to algae growth and cause eutrophication (Wang et al., 2016; Ge et al., 2017). In addition, phosphorus is a non-renewable resource mainly used as a nutrient in agricultural production (Montangero and Belevi, 2007). Due to an increase in the global demand for phosphorus resource, it will be depleted in the next 50–100 years (Takeda et al., 2010; Ye Y. Y. et al., 2017; Ye Z. L. et al., 2017). Therefore, the phosphorus recovery from wastewater is of considerable interest. However, a high solubility constant of the hypophosphite precipitants limits the transformation of hypophosphite into the precipitated products. In contrast, phosphate is easier to be recovered than hypophosphite by precipitation. As such, a highly efficient approach for the pre-oxidation of hypophosphite to phosphate becomes of great importance for phosphorus recovery. Photocatalysis, a nano-enabled technology, has been recognized for its promising applications with the generation of activated radicals such as hydroxyl radicals and superoxide anion radicals, which herein can be applied for hypophosphite oxidation.

Semiconductor photocatalysts have been recognized as a potential strategy to solve some serious challenges of the twenty-first century, such as energy shortage, environmental pollution, and global warming (Lin et al., 2017). TiO_2_ photocatalyst has attracted much attention due to its strong oxidizing power, low cost and high chemical stability. However, a large band gap (3.2 eV) of TiO_2_ means that it can only absorb ultraviolet light (only about 3–5% of total sunlight), which greatly limits its performance in industrial applications (Teng et al., 2017; Noman et al., 2018). Therefore, it is urgent to develop novel semiconductor photocatalysts that respond to visible light.

Recently, graphitic carbon nitride (g-C_3_N_4_), constituted by numerous layers of two-dimensional (2D) counterparts, has attracted enormous attention given its advantages such as low cost and visible light driven semiconductor photocatalyst (Huang et al., 2017b; Liu et al., 2017a,b; Tian et al., 2017; Wang et al., 2017). The metal free g-C_3_N_4_ can work as photocatalyst under visible light irradiation with a suitable band gap of 2.7 eV. In addition, g-C_3_N_4_ possesses excellently structural stability, which is suitable for chemical modification. Nevertheless, the photocatalytic property of g-C_3_N_4_ is still limited for its low surface area, low photoabsorption efficiency and high recombination rate of photo-induced electron-hole pairs (Jourshabani et al., 2017; Shao et al., 2017).

Decreasing the recombination of photo-induced carriers during the photocatalytic process would enhance the photocatalytic activity of the as-prepared g-C_3_N_4_ photocatalyst (Li J. D. et al., 2017). Therefore, some methods, such as porosity engineering, doping with foreign elements, and compositing with other semiconductors to facilitate charge separation, were developed to enhance the photocatalytic performance of g-C_3_N_4_. For example, the horn-like hollow mesoporous ultrathin g-C_3_N_4_ tubes were fabricated with high surface area, drastically boosted bulk charge separation, carrier density and surface charge transfer efficiency and showed the remarkably photocatalytic performance for H_2_ evolution (Liu et al., 2017a). Meanwhile, the 3D mesoporous g-C_3_N_4_ established by ultrathin self-doped nanosheets exhibited the superior photocatalytic performance on hydrogen evolution (Tian et al., 2017). Additionally, the porous and thin g-C_3_N_4_ nanosheets, prepared via a novel thiourea-assisted melamine-precursor hydrothermal pre-treatment followed by a traditional thermal polymerization method, profoundly enhanced visible-light photocatalytic performance on H_2_ evolution and NO removal from the gaseous phase (Huang et al., 2017b). Moreover, the Cl intercalated mesoporous g-C_3_N_4_ showed outstanding photocatalytic performance for water splitting into H_2_, CO_2_ reduction, liquid and air contaminants removal (Liu et al., 2017b).

Substoichiometric titanium oxides, known as Magnéli phases (Sun et al., 2016), comprise a series of compounds with the generic formula Ti_*n*_O_2*n*−1_ (4 ≤ *n* ≤ 10) (Kolbrecka and Przyluski, 1994; Guo et al., 2016). Among these compounds, Ti_4_O_7_ possesses high electrical conductivity, thermal stability, and corrosion resistance in harsh conditions (Oturan et al., 2017). Therefore, Ti_4_O_7_ is wildly used as catalyst coated material (Li et al., 2010), wastewater treatment material (You et al., 2016), support material in fuel cell (Chisaka et al., 2016), and additive to positive materials in batteries (Cao et al., 2017). However, it was also reported that pure Ti_4_O_7_ as the photocatalyst had limited photocatalytic activity with the band gap of 2.9 eV (Maragatha et al., 2017). Herein, coupling Ti_4_O_7_ and intrinsic g-C_3_N_4_ to construct the well-matched Ti_4_O_7_/g-C_3_N_4_ heterojunction would be an alternative novel pathway to address the intrinsic drawbacks of g-C_3_N_4_ for photocatalytic applications.

In this study, novel Ti_4_O_7_/g-C_3_N_4_ photocatalysts were synthesized at two different temperatures (100 and 160°C) and their performance in photocatalytic oxidation of hypophosphite under visible light irradiation and the corresponding mechanism were compared and investigated. Ti_4_O_7_/g-C_3_N_4_ photocatalysts exhibited an enhanced photocatalytic activity for hypophosphite oxidation under visible light irradiation and the annealing temperature significantly affected the photocatalytic performance. The separation mechanism of photogenerated electrons and holes of the photocatalysts was investigated by UV-Vis diffuse reflectance spectra, photoluminescence emission spectra, cyclic voltammetry (CV), and electrochemical impedance spectroscopy (EIS). The activated species generated in the photocatalytic process were measured by electron spin resonance (ESR). The enhanced photocatalytic performance could be ascribed to the efficient charge separation and transfer across the heterojunction interface and the enhanced photoabsorption efficiency. Our work demonstrated that the rational design and construction of isotype heterojunction was an effective strategy for the development of efficient photocatalysts under visible light irradiation.

## Materials and methods

### Chemicals

Melamine (C_3_H_6_N_6_), urea [CO(NH_2_)_2_], sub-stoichiometric titanium oxide (Ti_4_O_7_), sodium hypophosphite (NaH_2_PO_2_), sodium sulfate (Na_2_SO_4_), isopropanol [IPA, (CH_3_)_2_CHOH], ethylenediaminetetraacetic acid disodium salt (C_10_H_14_N_2_Na_2_O_8_), sodium hydroxide (NaOH), and sulfuric acid (H_2_SO_4_) were provided by Sinopharm Chemical Reagent Co., Ltd. (Beijing, China). 5,5-dimethylpyrroline-N-oxide (DMPO) was bought from Dojindo Co., Ltd. (Shanghai, China). The entire chemical reagents were analytical grade and all solutions were prepared using Milli-Q water (Millipore, 18.2 MΩ cm).

### Synthesis of g-C_3_N_4_ materials

The g-C_3_N_4_ materials were prepared using a liquid-based growth method (Sun et al., 2018). In a typical process, the mixture of melamine and urea (molar ratio = 1:1) was dissolved with 50 mL deionized water and then vigorously stirred for 1 h at room temperature. After that, the mixture suspension was centrifuged at 7,500 r/min for 15 min, and then dried at 60°C for 24 h under the vacuum to obtain the white powder. After that, the prepared white powders were further grinded into smaller powders in a mortar and placed in a muffle furnace. The powders were then annealed at 550°C in a muffle furnace for 4 h in static air at a ramp rate of 2.5°C min^−1^. The resulting yellow products were collected for further usage.

### Preparation of Ti_4_O_7_/g-C_3_N_4_ photocatalysts

The preparation procedure of Ti_4_O_7_/g-C_3_N_4_ photocatalysts was shown below: g-C_3_N_4_ powder (2.0 g) and Ti_4_O_7_ (1.0 g) were dispersed into 100 mL NaOH (0.1 mol/L) by ultrasonication for 0.5 h. Subsequently, the mixed liquor was transferred to reaction still and then annealed in different temperatures (100 and 160°C) for 20 h. After that, the obtained precipitates were collected by centrifugation and washed with distilled water, and then dried at 60°C for 12 h. Samples fabricated at different annealing temperatures were noted as 100-Ti_4_O_7_/g-C_3_N_4_ (fabricated at 100°C) and 160-Ti_4_O_7_/g-C_3_N_4_ (fabricated at 160°C), respectively.

### Samples characterization

The concentration of NaH_2_PO_2_ was measured by ion chromatography using a 732 IC detector (McDowell et al., 2004). The crystal phase composition and fineness of the samples were analyzed by X-ray diffraction (XRD) with Cu Kα radiation in the scanning range of 2θ = 5–80° (model D/max RA, Rigaku Co., Japan). The surface morphology of the as-developed samples was examined by scanning electron microscopy (SEM) (JEOL JSM-6701F). The valence state of the deposition was measured by X-ray photoelectron spectra (XPS) (PHI-5300/ESCA, ULVAC-PHI, INC). The UV–vis diffuse reflection spectra (UV–vis DRS) of the samples was obtained by a UV–vis spectrophotometer (UV-2450, Shimadzu, Japan). Electrochemical properties of the Ti_4_O_7_/g-C_3_N_4_ and g-C_3_N_4_ photocatalysts, including photocurrents (PC), CV, and EIS, were measured on a CHI 660B electrochemical system. Electron spin resonance (ESR) (ESRA-300, Bruker, Germany) signals were recorded by the probe molecular 5,5-dimethyl-1-pyrroline-N-oxide (DMPO) to identify the radicals generated under visible light irradiation (λ > 420 nm) (Tian et al., 2015).

### Evaluation of photocatalytic activity

The photocatalytic activities of as-synthesized samples were evaluated by the oxidation of hypophosphite in aqueous solution under visible light irradiation. For hypophosphite oxidation, the light source was a 35 W metal-halide lamp (Philips) with a 420 nm UV-cutoff filter. The lamps were located 12 cm away from the surface of reaction solution (about 5 mW cm^−2^). In each experiment, photocatalyst (10 mg) was dispersed in hypophosphite (100 ml, 100 mg L^−1^) aqueous solution. Before irradiation, the solution was continuously stirred in the dark for 2 h to reach adsorption-desorption equilibrium between the hypophosphite and the photocatalyst. During the photocatalytic reaction, the solutions were kept magnetically stirring, and 4 ml mixture was collected at 1 h intervals followed by centrifugation (10,000 rpm, 5 min) to remove the photocatalyst.

### Radicals quencher experiment for photocatalysis

In order to identify the contributions of the radicals generated in the photocatalytic oxidation process, IPA and N_2_ purging were applied with IPA acting as the ·OH radicals quencher and N_2_ purging reducing the superoxide ·O_2_^−^ radicals (Yang et al., 2016). Adding different radical scavengers into reaction solutions would affect the photocatalytic performance. As such, the contributions of ·OH radicals and ·O_2_^−^ radicals on photocatalytic oxidation of hypophosphite under visible light irradiation can be evaluated based on the change of photocatalytic oxidation efficiency of hypophosphite with and without IPA (1 mM) and N_2_ purging (continuous purging).

## Results and discussion

### Structure and morphology analyses

**Figure 1:**
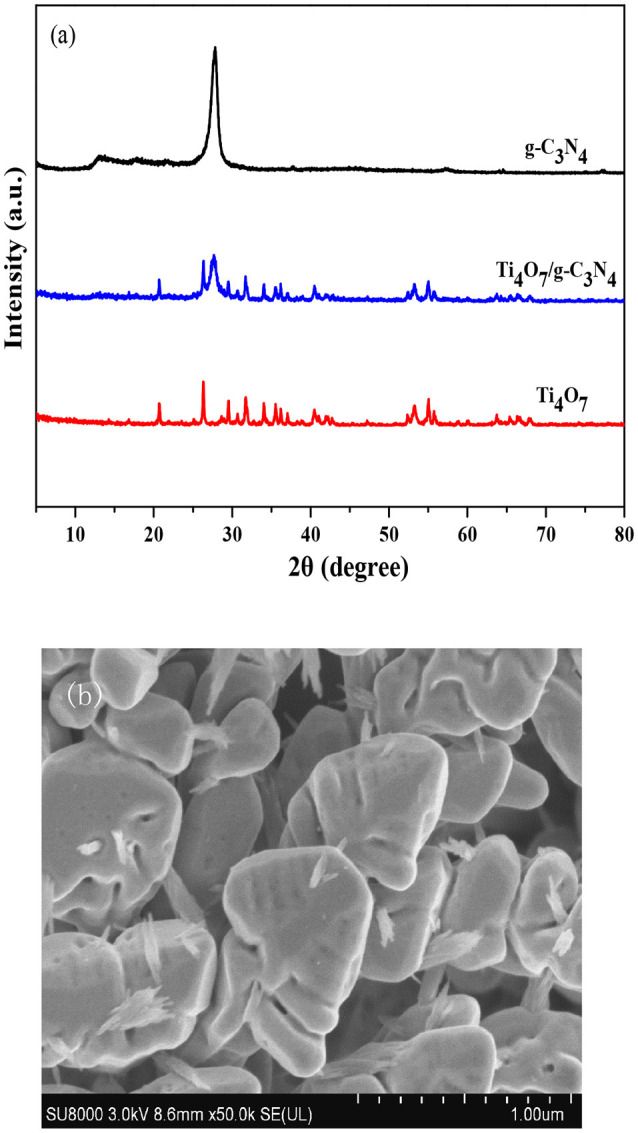
Structure and morphology analyses. **(a)** XRD patterns of the g-C_3_N_4_, Ti_4_O_7_ and 160-Ti_4_O_7_/g-C_3_N_4_ photocatalysts; **(b)** SEM image of the 160-Ti_4_O_7_/g-C_3_N_4_ photocatalyst.

The crystal structures of g-C_3_N_4_, Ti_4_O_7_, and 160-Ti_4_O_7_/g-C_3_N_4_ photocatalysts were characterized by XRD. As shown in Figure 1a, two pronounced diffraction peaks in pure g-C_3_N_4_ were observed at 13.20° and 27.60°, respectively. The peak at 13.20° was corresponded to (1 0 0) plane of tri-s-triazine units (Zhang et al., 2012a). The peak at 27.60°indexed as (0 0 2) peak was due to the interlayer-stacking of aromatic systems as in graphite (Zhang et al., 2012b). The characteristic peaks of Ti_4_O_7_ were matched well with the standard card (JCPDF 50-0787). The main diffraction peaks of 160-Ti_4_O_7_/g-C_3_N_4_ photocatalyst did not change obviously, indicating that the fabrication process did not destroy the main structure of both counterparts. The microstructure of the 160-Ti_4_O_7_/g-C_3_N_4_ photocatalyst was shown in Figure 1b. It was mainly composed of spheroidal crystals, and the shape of synthesized 160-Ti_4_O_7_/g-C_3_N_4_ photocatalyst was relatively uniform.

**Figure 2:**
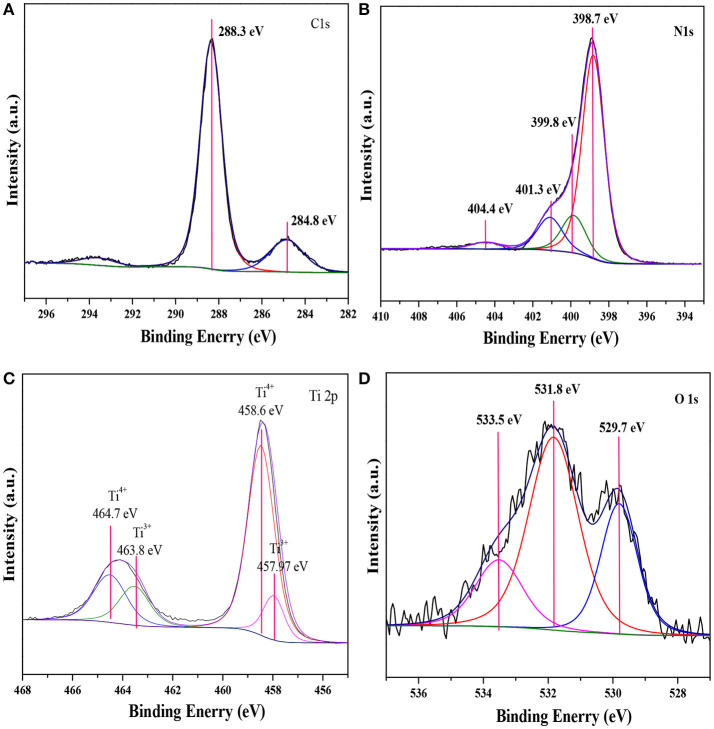
XPS spectra of the 160-Ti_4_O_7_/g-C_3_N_4_ photocatalyst. **(A)** C 1 s; **(B)** N 1 s; **(C)** Ti 2 p; **(D)** O 1 s.

XPS measurements were carried out to investigate the compositions and elemental chemical states of the samples. As shown in Figure 2, the XPS spectra revealed that the elements of C, N, Ti and O existed on the surface of 160-Ti_4_O_7_/g-C_3_N_4_ photocatalyst. The corresponding high resolution spectra of C 1s, N 1s, Ti 2p, and O 1s were also analyzed. The XPS spectra of C 1s core level for 160-Ti_4_O_7_/g-C_3_N_4_ photocatalyst was shown in Figure 2A that it could be divided into two components including the standard reference carbon (284.8 eV) and the sp^2^ bonded C in N = C(–N)_2_ (288.3 eV) (Jo and Natarajan, 2015). The N 1s spectra of Ti_4_O_7_/g-C_3_N_4_ could be divided into four peaks as shown in Figure 2B. The main peak at 398.7 eV was assigned to sp^2^ nitrogen (C = N–C) involved in triazine rings, and the peak at 399.8 eV originated from the tertiary nitrogen bonded to carbon atoms in the form of N–(C)_3_ (Wu et al., 2013). The peak at 401.3 eV was ascribed to amino functions (C–N–H). Another peak centered at 404.4 eV was associated to charging effects or positive charge localization in heterocycles (Gao et al., 2014). These assignments of C 1s and N 1s were agreed well with the XPS results of g-C_3_N_4_ reported previously. Ti_4_O_7_ is a mixed-valence compound with two evenly occupied Ti^4+^ (3d^0^) and Ti^3+^ (3d^1^) configurations. As shown in Figure 2C, two broad peaks at about 458.6 and 464.7 eV were observed, corresponding to the characteristic Ti 2p_1/2_ and Ti 2p_3/2_ peaks of Ti^4+^, respectively. Additionally, two peaks at 457.97 and 463.8 eV corresponding to Ti^3+^ also appeared, as reported elsewhere (Zeng et al., 2017). The O 1 s spectra of Ti_4_O_7_/g-C_3_N_4_ were shown in Figure 2D. The peak with binding energy of 533.5 eV was assigned to the C–O functional groups, and the peaks centered at the binding energies of 531.8 and 529.7 eV were ascribed to the OH–Ti and O–Ti bonds (Li Z. Q. et al., 2017). These results confirmed the presence of Ti_4_O_7_ on the g-C_3_N_4_ surface with covalent bonds.

### Photocatalytic activity analysis

**Figure 3:**
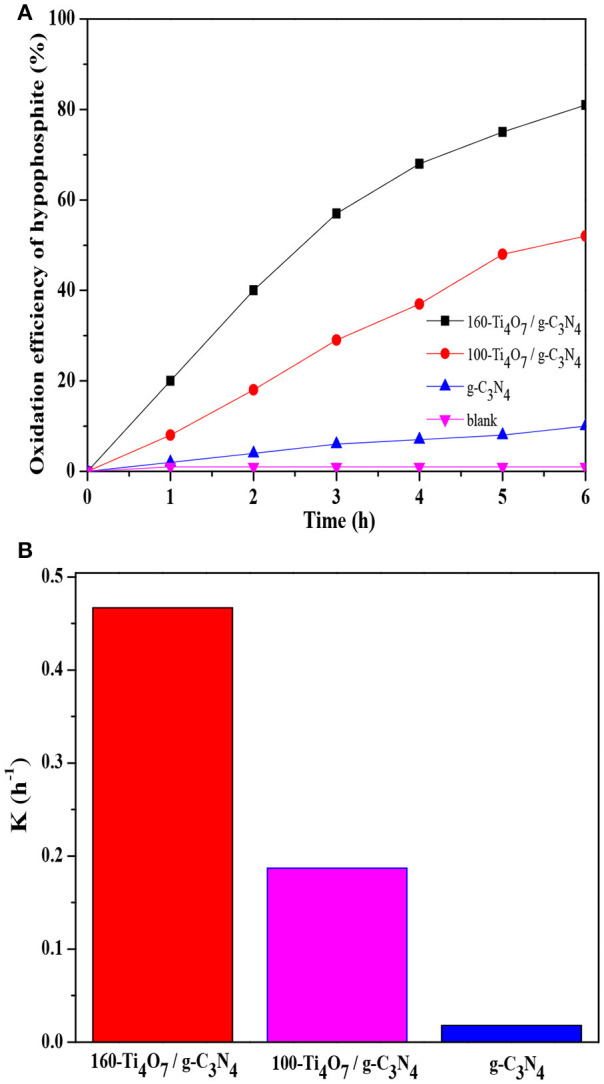
Photocatalytic activity of different photocatalysts. **(A)** The oxidation efficiency of hypophosphite; **(B)** The comparison of oxidation rate constant k.

The photocatalytic oxidation of hypophosphite over various samples was analyzed. As shown in Figure 3A, the blank experiment indicated that the concentration of hypophosphite was stable under visible light irradiation (λ > 420 nm) if there was no photocatalyst presented. Pure g-C_3_N_4_ showed weak photocatalytic activity with the oxidation efficiency of only 10% possibly owing to the rapid recombination of photo-generated charge carriers and low charge transfer ability (Shi et al., 2017). The 160-Ti_4_O_7_/g-C_3_N_4_ photocatalyst had the highest photocatalytic activity with the oxidation efficiency of 81% compared with the pure g-C_3_N_4_ and 100-Ti_4_O_7_/g-C_3_N_4_ photocatalysts.

The photocatalytic oxidation kinetic of the prepared samples was fitted by a pseudo-first-order model, which was depicted by the following Equation (1) (Lu et al., 2018):


(1) $$\text{ln }(C_{0}/C)=k\text{t}$$

Where *C*_0_ and *C* are the hypophosphite concentrations in solution at times 0 and *t*, respectively, and *k* is the first-order rate constant. As shown in Figure 3B, the 160-Ti_4_O_7_/g-C_3_N_4_ photocatalyst showed the highest photocatalytic oxidation rate of 0.467 h^−1^, which was 2.5 and 26 times higher than that of 100-Ti_4_O_7_/g-C_3_N_4_ and pure g-C_3_N_4_, respectively. Therefore, the results showed that the 160-Ti_4_O_7_/g-C_3_N_4_ photocatalyst exhibited an excellent activity in photocatalytic oxidation of hypophosphite under visible light irritation.

The enhanced photocatalytic activity of Ti_4_O_7_/g-C_3_N_4_ and the effect of annealing temperature on Ti_4_O_7_/g-C_3_N_4_ photocatalytic activity were further investigated and explained from the perspectives of photoabsorption efficiency, band gap, separation, transformation, and recombination processes of photogenerated carriers in the following sections.

### Optical properties analysis

**Figure 4:**
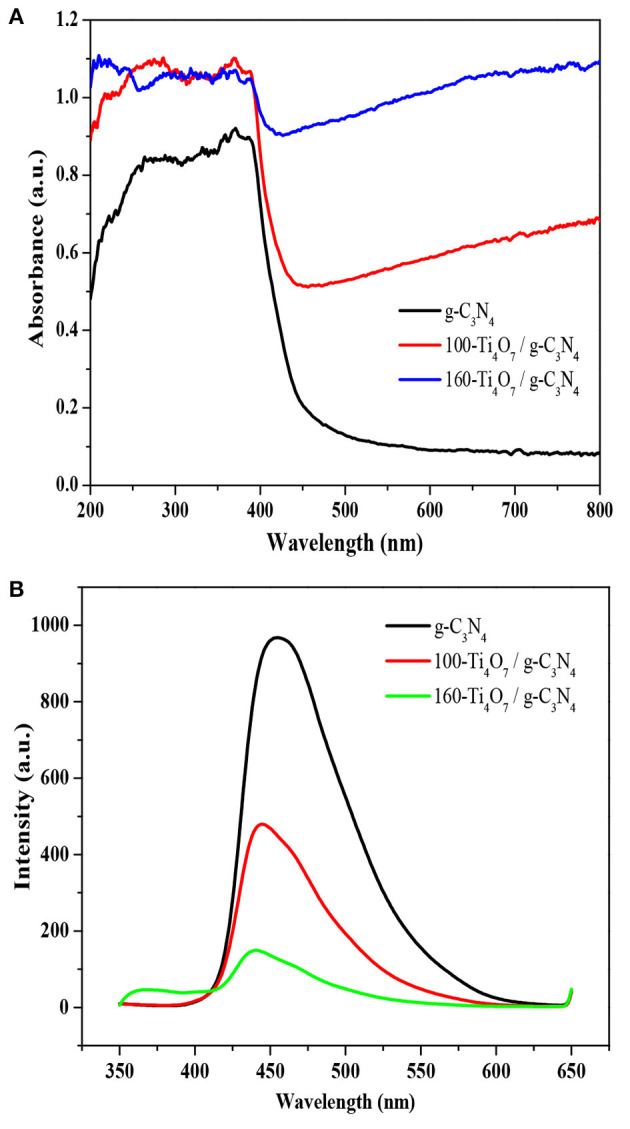
Optical properties of different photocatalysts. **(A)** UV–vis DRS spectra of the photocatalysts; **(B)** PL spectra of the photocatalysts.

The optical properties of Ti_4_O_7_/g-C_3_N_4_ and pure g-C_3_N_4_ photocatalysts were evaluated by UV–vis diffuse reflectance spectra. As shown in Figure 4A, the photoabsorption efficiency of Ti_4_O_7_/g-C_3_N_4_ was remarkably enhanced compared with the pure g-C_3_N_4_. The pure g-C_3_N_4_ held an absorption edge of around 430 nm while the Ti_4_O_7_/g-C_3_N_4_ photocatalysts showed a distinct red-shift, indicating that the Ti_4_O_7_/g-C_3_N_4_ photocatalysts were more efficient in light harvesting under visible light irradiation. The enhanced photoabsorption efficiency of Ti_4_O_7_/g-C_3_N_4_ was due to the narrowed band gap. The band energy gap of the photocatalysts was determined from the formula α*h*ν = *A (h*ν – *E*_*g*_*)*^*n*/2^ (*Eg*, α*, h*, ν*, and A* indicate the band gap, optical absorption coefficient, Plank's constant, photonic frequency and a proportionality constant, respectively) (Huang et al., 2017b; Liu et al., 2017a,b; Tian et al., 2017). The band gap calculated from the plot of absorption^1/2^ vs. energy was 2.70, 2.32, and 2.13 eV for g-C_3_N_4_, 100-Ti_4_O_7_/g-C_3_N_4_, and 160-Ti_4_O_7_/g-C_3_N_4_, respectively.

Photoluminescence (PL) spectra was used to investigate the separation, transformation and recombination processes of photogenerated carriers. The band–band PL spectrum can directly reflect the separation performance of photo-induced charge carriers, viz. the stronger of the band-band PL signal, the higher of the recombination rate of photo-induced carriers. The PL spectroscopy of the photocatalysts was shown in Figure 4B. All photocatalysts exhibited a broad emission peak centered at around 460 nm, which was mainly caused by the recombination of photogenerated electrons and holes produced by g-C_3_N_4_ (Shi et al., 2017). The PL emission intensity was highest for the pure g-C_3_N_4_, while the intensity was significantly lowered for Ti_4_O_7_/g-C_3_N_4_. This indicated that the charge carrier recombination was effectively suppressed for the Ti_4_O_7_/g-C_3_N_4_ photocatalysts. It is well known that the noble metals, such as Ti_4_O_7_, are good conductors with excellent electric properties. After formation of noble metals-semiconductors heterostructures, the photogenerated electrons of semiconductors could transfer through these noble metals rapidly and the lifetime of these electrons and holes are prolonged (Cui et al., 2017). In addition, the PL intensity of 160-Ti_4_O_7_/g-C_3_N_4_ was much lower than that of 100-Ti_4_O_7_/g-C_3_N_4_, indicating that 160-Ti_4_O_7_/g-C_3_N_4_ had the effectively decreased charge carrier recombination compared with 100-Ti_4_O_7_/g-C_3_N_4_. The higher annealing temperature could etch and tailor g-C_3_N_4_ with the possibly smaller and thinner nanosheet structure of 160-Ti_4_O_7_/g-C_3_N_4_ compared with 100-Ti_4_O_7_/g-C_3_N_4_ and thus shortened the distance between the photogenerated electrons and the heterostructure surface, which suppressed the recombination probability of photo-generated electron–hole pairs with the photogenerated electrons rapidly transferring through Ti_4_O_7_ (Dong et al., 2015).

### Electrochemical properties analysis

**Figure 5:**
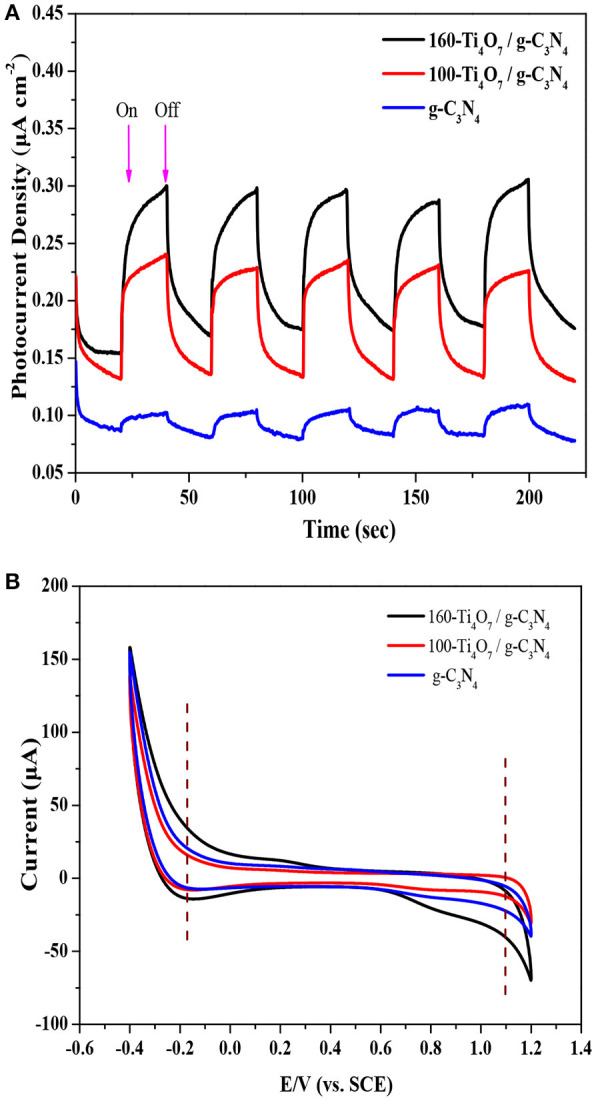
Electrochemical properties analysis of the 160-Ti_4_O_7_/g-C_3_N_4_ photocatalyst. **(A)** photocurrent density of different photocatalysts; **(B)** cyclic voltammogram scan of different photocatalysts.

The PC responses of g-C_3_N_4_, 100-Ti_4_O_7_/g-C_3_N_4_, and 160-Ti_4_O_7_/g-C_3_N_4_ photocatalysts under visible light irradiation were evaluated to further offer information about the separation and transformation efficiency of photogenerated electrons and holes. As shown in Figure 5A, the transient PC responses of all photocatalysts at light on and light off were reversible and stable, and the PC density of 160-Ti_4_O_7_/g-C_3_N_4_ was much higher (0.30 μA cm^−2^) than that of 100-Ti_4_O_7_/g-C_3_N_4_ (0.23 μA cm^−2^) and g-C_3_N_4_ (0.10 μA cm^−2^). This indicated that the Ti_4_O_7_/g-C_3_N_4_ heterostructures promoted the separation of photogenerated charge carriers (Kang et al., 2016), in line with the PL spectra results as shown in Figure 4B. Additionally, this was also supported by the CV test. As shown in Figure 5B, a reduction peak at about −0.18 V was observed in g-C_3_N_4_, 100-Ti_4_O_7_/g-C_3_N_4_, and 160-Ti_4_O_7_/g-C_3_N_4_, but 160-Ti_4_O_7_/g-C_3_N_4_ photocatalyst possessed much higher reduction current, which indicated faster electron transfer in 160-Ti_4_O_7_/g-C_3_N_4_ photocatalyst (Samanta and Srivastava, 2017).

**Figure 6:**
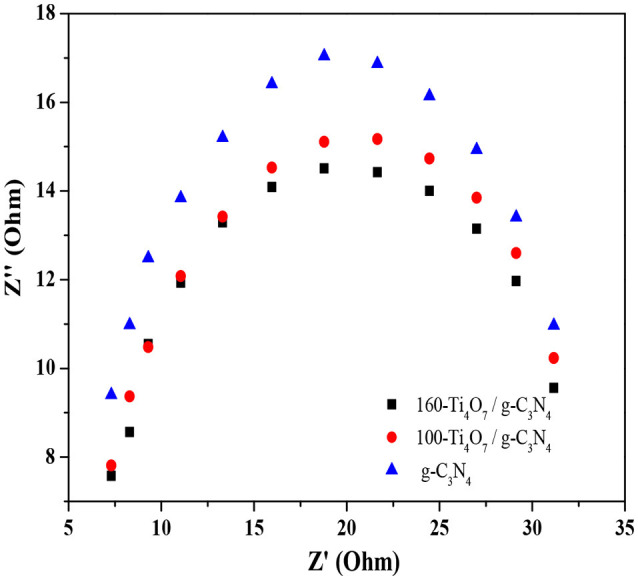
EIS Nyquist plots of the g-C_3_N_4_, 100-Ti_4_O_7_/g-C_3_N_4_, and 160-Ti_4_O_7_/g-C_3_N_4_ photocatalysts under visible light irradiation.

Electrochemical impedance spectroscopy was used to investigate the photogenerated charge separation process on the photocatalysts. The radius of the circular arc reflected the resistance of the interfacial charge transfer and separation efficiency of the electron-hole pairs (Leng et al., 2005; Liang and Zhu, 2016). As shown in Figure 6, the arc radius decreased gradually when Ti_4_O_7_ was doped onto the g-C_3_N_4_ photocatalyst. This meant that the photogenerated charge separation process occurred more easily on Ti_4_O_7_/g-C_3_N_4_ compared with the pure g-C_3_N_4_ because of the decreased energy barrier that the electrode reaction needed to overcome. Moreover, the arc radius of 160-Ti_4_O_7_/g-C_3_N_4_ was smaller than that of 100-Ti_4_O_7_/g-C_3_N_4_, meaning that the separation of the photogenerated electron–hole pairs was more effective and the interfacial charge transfer of the electron donor/electron acceptor was faster on the 160-Ti_4_O_7_/g-C_3_N_4_ photocatalyst.

### Catalyst stability analysis

**Figure 7:**
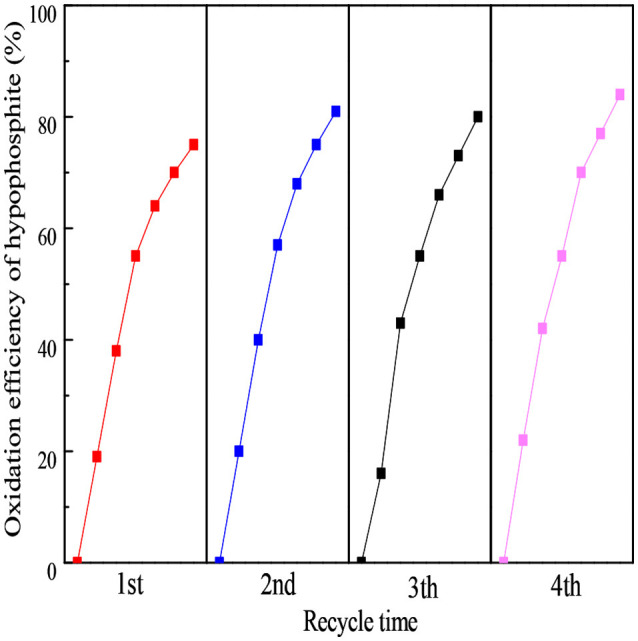
The stability analysis of the 160-Ti_4_O_7_/g-C_3_N_4_ photocatalyst under repetitive experiments.

The stability was another vital consideration for an excellent photocatalyst. To evaluate the stability of the as-prepared 160-Ti_4_O_7_/g-C_3_N_4_ photocatalyst, the repetitive experiments of photocatalytic oxidation of hypophosphite were carried out. As shown in Figure 7, the oxidation efficiency of hypophosphite in the four-round continuous reaction tests using 160-Ti_4_O_7_/g-C_3_N_4_ photocatalyst was 75, 81, 80, and 84%, respectively. The repetitive experiments results showed that the fabricated Ti_4_O_7_/g-C_3_N_4_ photocatalysts had a stable structure possibly with the strong binding force. The N-H groups or conjugated structures in g-C_3_N_4_ could tightly bond with Ti^4+^ (3d^0^) and Ti^3+^ (3d^1^) in Ti_4_O_7_, which effectively reduced the dissolution of bulk g-C_3_N_4_ material during the photocatalytic process.

### Proposed mechanism

**Figure 8:**
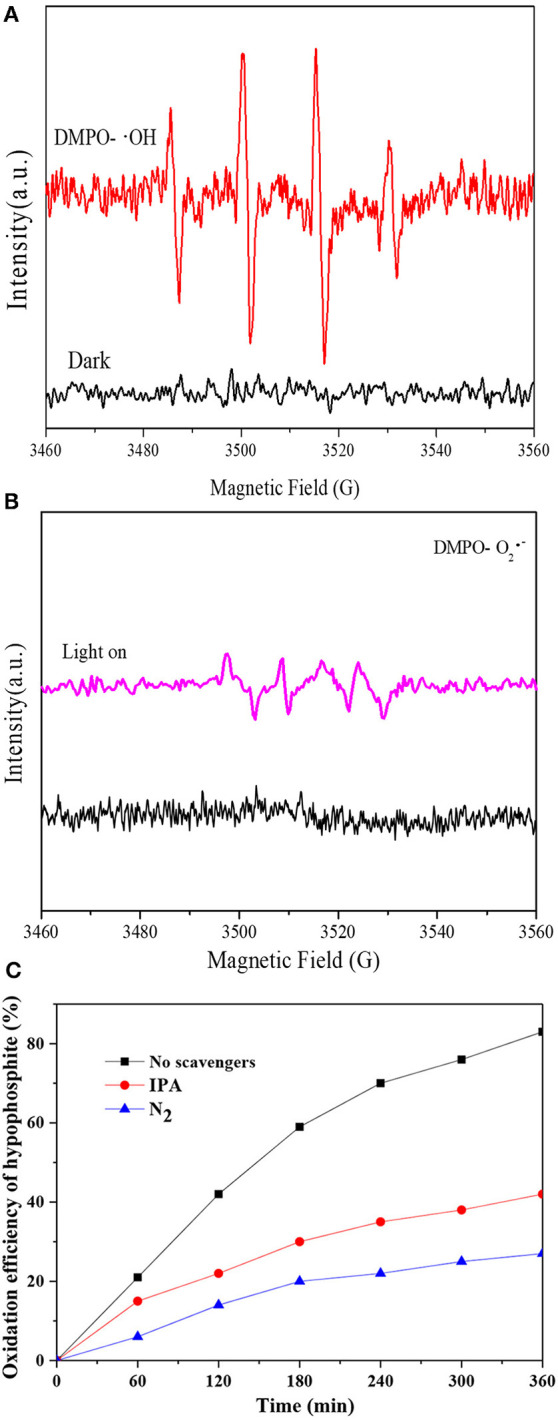
Radials analyses. **(A)** DMPO spin-trapping ESR spectra for ·OH radials analysis; **(B)** DMPO spin-trapping ESR spectra for ·O_2_^−^ radials analysis; **(C)** Effect of scavengers on the photocatalytic oxidation process.

To clarify the reaction mechanism of photocatalytic oxidation of hypophosphite, the ROS generated under visible light irradiation were analyzed by ESR technique (with DMPO). As shown in Figure 8A, no ESR signals were observed in the dark while ·OH was observed under visible light irradiation with four peaks with intensities of 1:2:2:1 attributing to DMPO-·OH generated via a hole oxidative process on H_2_O and/or OH^−^ (Tu et al., 2017). Moreover, ·O_2_^−^ was also observed under visible light irradiation with a four-line spectrum with the relative intensities of 1:1:1:1 assigned to DMPO-·O_2_^−^ adduct derived from O_2_ reduction by electrons (Huang et al., 2015), however, no ESR signals were observed in the dark as shown in Figure 8B. Therefore, both ·OH and ·O_2_^−^ would contribute to the photocatalytic oxidation of hypophosphite and their contributions were further investigated with and without radical scavengers. Isopropanol (IPA) and N_2_ purging were applied with IPA acting as the ·OH radicals quencher and N_2_ purging reducing the superoxide ·O_2_^−^ radicals (Yang et al., 2016). As shown in Figure 8C, the photocatalytic oxidation efficiency of hypophosphite decreased from 83% (without radical scavengers) to 42% (with IPA) and even 27% (with N_2_ purging). These results confirmed that both ·OH and O_2_·^−^ radicals were the major active radical species for hypophosphite oxidation in the photocatalytic process with O_2_·^−^ accounting for a more significant contribution. Note that the photocatalytic oxidation efficiency of hypophosphite did not decrease to zero with the lowest efficiency of 27% in the presence of N_2_ purging, indicating that holes and some other radicals may also contribute to the photocatalytic oxidation process to some extent. It was reported that reactive oxygen species (ROS), such as superoxide (·O_2_^−^), hydroxyl radicals (·OH), singlet oxygen (^1^O_2_), peroxyl (RO_2_·), and alkoxyl (RO-) as well as hypochlorous acid (HOCl) are basically produced in the photocatalytic process (Huang et al., 2017a), which may also contribute to the photocatalytic oxidation of hypophosphite in this case.

**Figure 9:**
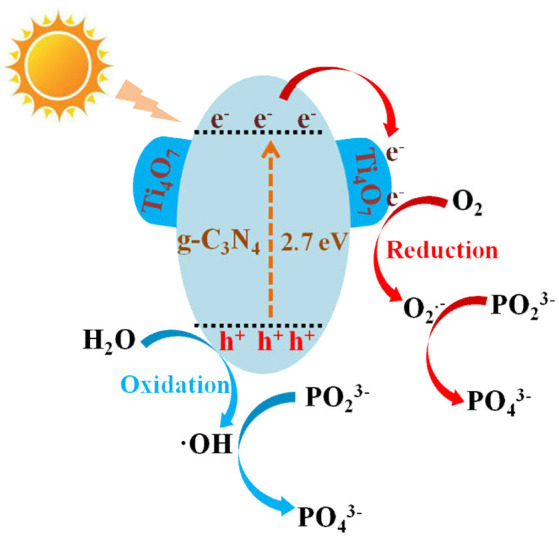
The mechanism of the hypophosphite oxidation over the Ti_4_O_7_/g-C_3_N_4_ photocatalyst under visible light irradiation.

According to the above results and those reported in the literature, the possible photocatalytic mechanism of Ti_4_O_7_/g-C_3_N_4_ on hypophosphite oxidation was illustrated in Figure 9. The possible photocatalytic processes were as follows:


(2) $${\text{Ti}}_{4}{\text{O}}_{7}/\text{g}-{\text{C}}_{3}{\text{N}}_{4}+hv\to \text{g}-{\text{C}}_{3}{\text{N}}_{4}({\text{e}}^{-}+{\text{h}}^{+})$$

(3) $$\text{g}-{\text{C}}_{3}{\text{N}}_{4}\;({\text{e}}^{-})+{\text{Ti}}_{4}{\text{O}}_{7}\to {\text{Ti}}_{4}{\text{O}}_{7}\;({\text{e}}^{-})$$

(4) $${\text{Ti}}_{4}{\text{O}}_{7}+hv\to {\text{Ti}}_{4}{\text{O}}_{7}\;({\text{e}}^{-})$$

(5) $${\text{Ti}}_{4}{\text{O}}_{7}\;({\text{e}}^{-})+{\text{O}}_{2}\to \cdot {\text{O}}_{2}^{-}$$

(6) $$\cdot {\text{O}}_{2}^{-}+{\text{PO}}_{2}^{3-}\to {\text{PO}}_{4}^{3-}$$

(7) $$\text{g}-{\text{C}}_{3}{\text{N}}_{4}\;({\text{h}}^{+})+{\text{OH}}^{-}\to \cdot \text{OH}$$

(8) $$\cdot \text{OH}+{\text{PO}}_{2}^{3-}\to {\text{PO}}_{4}^{3-}$$

(9) $$\text{g}-{\text{C}}_{3}{\text{N}}_{4}\;({\text{h}}^{+})+{\text{PO}}_{2}^{3-}\;\to {\text{ PO}}_{4}^{3-}$$

Firstly, the electrons (e^−^) in valence band of g-C_3_N_4_ under visible light irradiation could be excited to the conduction band, leaving the holes (h^+^) in valence band of g-C_3_N_4_ (Equation 2). Then the photogenerated electrons in the conduction band of g-C_3_N_4_ would continually transfer to Ti_4_O_7_ until the same Fermi levels reached (Equation 3). Thus, the photogenerated electrons and holes were located at Ti_4_O_7_ and g-C_3_N_4_, respectively, leading to the effective separation of the photoinduced charge carriers. Furthermore, the electrons might be also generated from the Ti_4_O_7_ particles (Equation 4) with the product easily reacting with the adsorbed oxygen molecules to produce ·${\text{O}}_{2}^{-}$ (Equation 5) followed by the oxidation of hypophosphite to phosphate (Equation 6). Meanwhile, the photogenerated holes as the strong oxidants could oxidize OH^−^ to ·OH radicals, and then the hypophosphite was directly oxidized to phosphate (Equations 7 and 8). In addition, some photogenerated holes could directly oxidize hypophosphite to phosphate (Equation 9).

## Conclusion

The enhancement of Ti_4_O_7_/g-C_3_N_4_ visible light photocatalytic performance on hypophosphite oxidation and the effect of annealing temperature and the corresponding mechanism were investigated in this study. 160-Ti_4_O_7_/g-C_3_N_4_ (fabricated at 160°C) photocatalyst showed the highest oxidation efficiency of hypophosphite of 81% and the highest photocatalytic oxidation rate of 0.467 h^−1^ comparing with 100-Ti_4_O_7_/g-C_3_N_4_ (fabricated at 100°C) and pure g-C_3_N_4_. The enhanced photocatalytic performance of 160-Ti_4_O_7_/g-C_3_N_4_ could be ascribed to the effective charge separation and enhanced photoabsorption efficiency. Additionally, ESR results showed that hydroxyl radicals and superoxide anion radicals were mainly responsible to the oxidation of hypophosphite with O_2_·^−^ accounting for a more significant contribution. Moreover, Ti_4_O_7_/g-C_3_N_4_ photocatalysts showed the remarkable stability in the repetitive experiments. Our work demonstrated that the rational design and construction of isotype heterojunction was an effective strategy to develop the efficient photocatalysts under visible light irradiation.

## Author contributions

All authors listed have made a substantial, direct and intellectual contribution to the work, and approved it for publication.

### Conflict of interest statement

Author LY was employed by company of Heibei Yinfa Meifute Environmental Engineering Co., Ltd. The other authors declare that the research was conducted in the absence of any commercial or financial relationships that could be construed as a potential conflict of interest.

## References

[B1] Bulasara V. K. Thakuria H. Uppaluri R. Purkait M. K. (2011). Effect of process parameters on electroless plating and nickel-ceramic composite membrane characteristics. Desalination 268, 195–203. 10.1016/j.desal.2010.10.025

[B2] Cao X. C. Sun Z. H. Zheng X. J. Tian J. H. Jin C. Yang R. Z.. (2017). MnCo_2_O_4_ decorated Magnéli phase titanium oxide as a carbon-free cathode for Li–O_2_ batteries. J. Mater. Chem. A 5, 19991–19996. 10.1039/c7ta06152h

[B3] Chisaka M. Ando Y. Yamamoto Y. Itagaki N. (2016). A carbon-support-free titanium oxynitride catalyst for proton exchange membrane fuel cell cathodes. Electrochim. Acta 214, 165–172. 10.1016/j.electacta.2016.08.032

[B4] Cui W. Li J. Y. Cen W. L. Sun Y. J. Lee S. C. Dong F. (2017). Steering the interlayer energy barrier and charge flow via bioriented transportation channels in g-C_3_N_4_: enhanced photocatalysis and reaction mechanism. J. Catal. 352, 351–360. 10.1016/j.jcat.2017.05.017

[B5] Dong F. Li Y. H. Wang Z. Y. Ho W. K. (2015). Enhanced visible light photocatalytic activity and oxidation ability of porous graphene-like g-C_3_N_4_ nanosheets via thermal exfoliation. Appl. Surf. Sci. 358, 393–403. 10.1016/j.apsusc.2015.04.034

[B6] Gao D. Q. Xu Q. Zhang J. Yang Z. L. Si M. S. Yan Z. J.. (2014). Defect-related ferromagnetism in ultrathin metal-free C_3_N_4_ nanosheets. Nanoscale 6, 2577–2581. 10.1039/c3nr04743a 24464248

[B7] Ge C. Chai Y. Wang H. Kan M. (2017). Ocean acidification: one potential driver of phosphorus eutrophication. Mar. Pollut. Bull. 115, 149–153. 10.1016/j.marpolbul.2016.12.016 27979616

[B8] Guo L. Jing Y. Chaplin B. P. (2016). Development and characterization of ultrafiltration TiO_2_ magnéli phase reactive electrochemical membranes. Environ. Sci. Technol. 50, 1428–1436. 10.1021/acs.est.5b04366 26735740

[B9] Huang H. Han X. Li X. Wang S. Chu P. Zhang Y. (2015). Fabrication of multiple heterojunctions with tunable visible-light-active photocatalytic reactivity in BiOBr–BiOI full-range composites based on microstructure modulation and band structures. ACS Appl. Mater. Interfaces 7, 482–492. 10.1021/am5065409 25525911

[B10] Huang H. Tu S. Zeng C. Zhang T. Reshak A. Zhang Y. H. (2017a). Macroscopic polarization enhancement promoting photo- and piezoelectric-induced charge separation and molecular oxygen activation. Angew. Chem. Int. Edn. 56, 11860–11864. 10.1002/anie.201706549 28731229

[B11] Huang H. W. Xiao K. Tian N. Dong F. Zhang T. R. Du X.. (2017b). Template-free precursor-surface-etching route to porous, thin g-C_3_N_4_ nanosheets for enhancing photocatalytic reduction and oxidation activity. J. Mater. Chem. A 5, 17452–17463. 10.1039/c7ta04639a

[B12] Jo W. K. Natarajan T. S. (2015). Influence of TiO_2_ morphology on the photocatalytic efficiency of direct Z-scheme g-C_3_N_4_/TiO_2_ photocatalysts for isoniazid degradation. Chem. Eng. J. 281, 549–565. 10.1016/j.cej.2015.06.120

[B13] Jourshabani M. Shariatinia Z. Badiei A. (2017). Facile one-pot synthesis of cerium oxide/sulfur-doped graphitic carbon nitride (g-C_3_N_4_) as efficient nanophotocatalysts under visible light irradiation. J. Colloid Interface Sci. 507, 59–73. 10.1016/j.jcis.2017.07.106 28780336

[B14] Kang K. Watanabe S. Broch K. Sepe A. Brown A. Nasrallah I.. (2016). 2D coherent charge transport in highly ordered conducting polymers doped by solid state diffusion. Nat. Mater. 15, 896–902. 10.1038/NMAT4634 27159015

[B15] Kolbrecka K. Przyluski J. (1994). Sub-stoichiometric titanium oxides as ceramic electrodes for oxygen evolution—structural aspects of the voltammetric behaviour of Ti_n_O_2n−1_. Electrochim. Acta 39, 1591–1595. 10.1016/0013-4686(94)85140-9

[B16] Leng W. H. Zhang Z. Zhang J. Q. Cao C. N. (2005). Investigation of the kinetics of a TiO_2_ photoelectrocatalytic reaction involving charge transfer and recombination through surface states by electrochemical impedance spectroscopy. J. Phys. Chem. B 109, 15008–15023. 10.1021/jp051821z 16852900

[B17] Li J. D. Zhang X. L. Raziq F. Wang J. S. Liu C. Liu Y. D.. (2017). Improved photocatalytic activities of g-C_3_N_4_ nanosheets by effectively trapping holes with halogen-induced surface polarization and 2,4-dichlorophenol decomposition mechanism. Appl. Catal. B Environ. 218, 60–67. 10.1016/j.apcatb.2017.06.038

[B18] Li L. Y. Takahashi N. Kaneko K. Shimizu T. Takarada T. (2015). A novel method for nickel recovery and phosphorus removal from spent electroless nickel-plating solution. Sep. Purif. Technol. 147, 237–244. 10.1016/j.seppur.2015.04.029

[B19] Li X. X. Zhu A. L. Qu W. Wang H. J. Hui R. Zhang L.. (2010). Magneli phase Ti_4_O_7_ electrode for oxygen reduction reaction and its implication for zinc-air rechargeable batteries. Electrochim. Acta 55, 5891–5898. 10.1016/j.electacta.2010.05.041

[B20] Li Z. Q. Qi M. Y. Tu C. Y. Wang W. P. Chen J. R. Wang A. J. (2017). Highly efficient removal of chlorotetracycline from aqueous solution using graphene oxide/TiO_2_ composite: properties and mechanism. Appl. Surf. Sci. 425, 765–775. 10.1016/j.apsusc.2017.07.027

[B21] Liang F. F. Zhu Y. F. (2016). Enhancement of mineralization ability for phenol via synergetic effect of photoelectrocatalysis of g-C_3_N_4_ film. Appl. Catal. B Environ. 180, 324–329. 10.1016/j.apcatb.2015.05.009

[B22] Lin F. Shao B. Li Z. Zhang J. Y. Wang H. Zhang S. H.. (2017). Visible light photocatalysis over solid acid: enhanced by gold plasmonic effect. Appl. Catal. B Environ. 218, 480–487. 10.1016/j.apcatb.2017.06.076

[B23] Liu C. Y. Huang H. W. Ye L. Q. Yu S. X. Tian N. Du X.. (2017a). Intermediate-mediated strategy to horn-like hollow mesoporous ultrathin g-C_3_N_4_ tube with spatial anisotropic charge separation for superior photocatalytic H_2_ evolution. Nano Energy 41, 738–748. 10.1016/j.nanoen.2017.10.031

[B24] Liu C. Y. Zhang Y. H. Dong F. Reshak A. H. Ye L. Q. Pinna N.. (2017b). Chlorine intercalation in graphitic carbon nitride for efficient photocatalysis. Appl. Catal. B Environ. 203, 465–474. 10.1016/j.apcatb.2016.10.002

[B25] Lu X. J. Wang Y. Zhang X. Y. Xu G. Q. Wang D. M. Lv J.. (2018). NiS and MoS_2_ nanosheet co-modified graphitic C_3_N_4_ ternary heterostructure for high efficient visible light photodegradation of antibiotic. J. Hazard. Mater. 341, 10–19. 10.1016/j.jhazmat.2017.07.004 28763632

[B26] Maragatha J. Rani C. Rajendran S. Karuppuchamy S. (2017). Microwave synthesis of nitrogen doped Ti_4_O_7_ for photocatalytic applications. Physica E 93, 78–82. 10.1016/j.physe.2017.05.020

[B27] McDowell M. M. Ivey M. M. Lee M. E. Firpo V. V. Salmassi T. M. Khachikian C. S.. (2004). Detection of hypophosphite, phosphite, and orthophosphate in natural geothermal water by ion chromatography. J. Chromatogr. A 1039, 105–111. 10.1016/j.chroma.2003.11.056 15250410

[B28] Montangero A. Belevi H. (2007). Assessing nutrient flows in septic tanks by eliciting expert judgement: a promising method in the context of developing countries. Water Res. 41, 1052–1064. 10.1016/j.watres.2006.10.036 17223156

[B29] Noman M. T. Wiener J. Saskova J. Ashraf M. A. Vikova M. Jamshaid H.. (2018). *In-situ* development of highly photocatalytic multifunctional nanocomposites by ultrasonic acoustic method. Ultrason. Sonochem. 40, 41–56. 10.1016/j.ultsonch.2017.06.026 28946440

[B30] Oturan N. Ganiyu S. O. Raffy S. Oturan M. A. (2017). Sub-stoichiometric titanium oxide as a new anode material for electro-Fenton process: application to electrocatalytic destruction of antibiotic amoxicillin. Appl. Catal. B Environ. 217, 214–223. 10.1016/j.apcatb.2017.05.062

[B31] Samanta S. Srivastava R. (2017). Thermal catalysis vs. photocatalysis: a case study with FeVO_4_/g-C_3_N_4_ nanocomposites for the efficient activation of aromatic and benzylic C–H bonds to oxygenated products. Appl. Catal. B Environ. 218, 621–636. 10.1016/j.apcatb.2017.06.043

[B32] Shao H. X. Zhao X. Wang Y. B. Mao R. Wang Y. Qiao M.. (2017). Synergetic activation of peroxymonosulfate by Co_3_O_4_ modified g-C_3_N_4_ for enhanced degradation of diclofenac sodium under visible light irradiation. Appl. Catal. B Environ. 218, 810–818. 10.1016/j.apcatb.2017.07.016

[B33] Shi A. Y. Li H. H. Yin S. Liu B. Zhang J. C. Wang Y. H. (2017). Effect of conjugation degree and delocalized π-system on the photocatalytic activity of single layer g-C_3_N_4_. Appl. Catal. B Environ. 218, 137–146. 10.1016/j.apcatb.2017.06.017

[B34] Sun S. P. Liao X. M. Yin G. F. Yao Y. D. Huang Z. B. Pu X. M. (2016). Enhanced electrochemical performance of TiO_2_ nanotube array electrodes by controlling the introduction of substoichiometric titanium oxides. J. Alloy. Compd. 680, 538–543. 10.1016/j.jallcom.2016.04.171

[B35] Sun X. Jiang D. Zhang L. Wang W. Z. (2018). Alkaline modified g-C_3_N_4_ photocatalyst for high selective oxide coupling of benzyl alcohol to benzoin. Appl. Catal. B Environ. 220, 553–560. 10.1016/j.apcatb.2017.08.057

[B36] Takeda I. Somura H. Mori Y. (2010). Recovery of phosphorus from natural water bodies using iron-oxidizing bacteria and woody biomass. Ecol. Eng. 36, 1064–1069. 10.1016/j.ecoleng.2010.04.019

[B37] Teng W. Wang Y. M. Huang H. H. Li X. Y. Tang Y. B. (2017). Enhanced photoelectrochemical performance of MoS_2_ nanobelts-loaded TiO_2_ nanotube arrays by photo-assisted electrodeposition. Appl. Surf. Sci. 425, 507–517. 10.1016/j.apsusc.2017.06.297

[B38] Tian N. Zhang Y. H. Li X. W. Xiao K. Du X. Dong F.. (2017). Precursor-reforming protocol to 3D mesoporous g-C_3_N_4_ established by ultrathin self-doped nanosheets for superior hydrogen evolution. Nano Energy 38, 72–81. 10.1016/j.nanoen.2017.05.038

[B39] Tian S. C. Li Y. B. Zhao X. (2015). Cyanide removal with a copper/active carbon fiber cathode via a combined oxidation of a Fenton-like reaction and *in situ* generated copper oxides at anode. Electrochim. Acta 180, 746–755. 10.1016/j.electacta.2015.09.006

[B40] Tu S. C. Huang H. W. Zhang T. R. Zhang Y. H. (2017). Controllable synthesis of multi-responsive ferroelectric layered perovskite-like Bi_4_Ti_3_O_12_: photocatalysis and piezoelectric-catalysis and mechanism insight. Appl. Catal. B Environ. 219, 550–562. 10.1016/j.apcatb.2017.08.001

[B41] Wang D. Chen N. Yu Y. Hu W. W. Feng C. P. (2016). Investigation on the adsorption of phosphorus by Fe-loaded ceramic adsorbent. J. Colloid Interface Sci. 464, 277–284. 10.1016/j.jcis.2015.11.039 26624533

[B42] Wang W. Fang J. J. Shao S. F. Lai M. Lu C. H. (2017). Compact and uniform TiO_2_@g-C_3_N_4_ core-shell quantum heterojunction for photocatalytic degradation of tetracycline antibiotics. Appl. Catal. B Environ. 217, 57–64. 10.1016/j.apcatb.2017.05.037

[B43] Wu G. S. Thind S. S. Wen J. L. Yan K. Chen A. C. (2013). A novel nanoporous α-C_3_N_4_ photocatalyst with superior high visible light activity. Appl. Catal. B Environ. 142–143, 590–597. 10.1016/j.apcatb.2013.05.070

[B44] Yang X. L. Qian F. F. Zou G. J. Li M. L. Lu J. R. Li Y. M.. (2016). Facile fabrication of acidified g-C_3_N_4_/g-C_3_N_4_ hybrids with enhanced photocatalysis performance under visible light irradiation. Appl. Catal. B Environ. 193, 22–35. 10.1016/j.apcatb.2016.03.060

[B45] Ye Y. Y. Ngo H. H. Guo W. S. Liu Y. W. Li J. X. Liu Y.. (2017). Insight into chemical phosphate recovery from municipal wastewater. Sci. Total Environ. 576, 159–171. 10.1016/j.scitotenv.2016.10.078 27783934

[B46] Ye Z. L. Deng Y. Y. Lou Y. Y. Ye X. Zhang J. Q. Chen S. H. (2017). Adsorption behavior of tetracyclines by struvite particles in the process of phosphorus recovery from synthetic swine wastewater. Chem. Eng. J. 313, 1633–1638. 10.1016/j.cej.2016.11.062

[B47] You S. J. Liu B. Gao Y. F. Wang Y. Tang C. Y. Y. Huang Y. B.. (2016). Monolithic porous magnéli-phase Ti_4_O_7_ for electro-oxidation treatment of industrial wastewater. Electrochim. Acta 214, 326–335. 10.1016/j.electacta.2016.08.037

[B48] Zeng X. K. Wang Z. Y. Wang G. Gengenbach T. R. McCarthy D. T. Deletic A.. (2017). Highly dispersed TiO_2_ nanocrystals and WO_3_ nanorods on reduced graphene oxide: Z-scheme photocatalysis system for accelerated photocatalytic water disinfection. Appl. Catal. B Environ. 218, 163–173. 10.1016/j.apcatb.2017.06.055

[B49] Zhang J. S. Zhang G. G. Chen X. F. Lin S. Möhlmann L. Dołega G.. (2012a). Co-monomer control of carbon nitride semiconductors to optimize hydrogen evolution with visible light. Angew. Chem. Int. Ed. 124, 3237–3241. 10.1002/anie.201106656 22334504

[B50] Zhang J. S. Zhang M. W. Zhang G. G. Wang X. C. (2012b). Synthesis of carbon nitride semiconductors in sulfur flux for water photoredox catalysis. ACS Catal. 2, 940–948. 10.1021/cs300167b

