# Corrigendum: Ti_4_O_7_/g-C_3_N_4_ visible light photocatalytic performance on hypophosphite oxidation: Effect of annealing temperature

**DOI:** 10.3389/fchem.2022.1114074

**Published:** 2023-01-18

**Authors:** Wei Guan, Gaoge Sun, Lei Yin, Zhenghua Zhang, Shichao Tian

**Affiliations:** ^1^ Chongqing Key Laboratory of Environmental Materials and Remediation Technologies, Chongqing University of Arts and Sciences, Chongqing, China; ^2^ Department of Chemistry, Tsinghua University, Beijing, China; ^3^ Heibei Yinfa Meifute Environmental Engineering Co., Ltd., Shijiazhuang, China; ^4^ Research Institute of Environmental Engineering and Nano-Technology, Graduate School at Shenzhen, Tsinghua University, Shenzhen, China; ^5^ Shenzhen Environmental Science and New Energy Technology Engineering Laboratory, Tsinghua-Berkeley Shenzhen Institute, Shenzhen, China

**Keywords:** graphitic carbon nitride, sub-stoichiometric titanium oxides, hypophosphite, hydroxyl radicals, superoxide anion radicals

In the original article, there was an error in Figure 8A as published. The corrected Figure 8 and its caption appear below.

**FIGURE 8:**
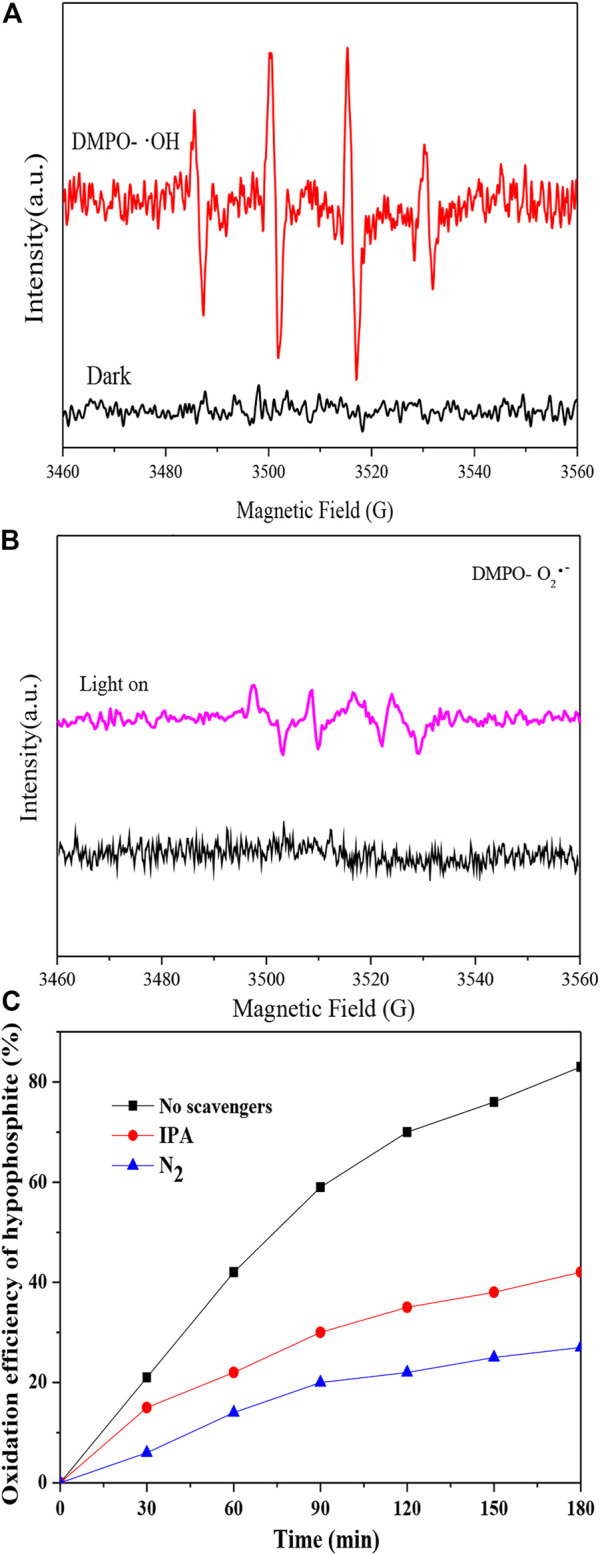
Radials analyses: **(A)** DMPO spin-trapping ESR spectra for **·**OH radials analysis; **(B)** DMPO spin-trapping ESR spectra for **·**O_2_^−^ radials analysis; **(C)** Effect of scavengers on the photocatalytic oxidation process.

The authors apologize for this error and state that this does not change the scientific conclusions of the article in any way. The original article has been updated.

